# Navigating the Complex Terrain of Dysregulated Microglial Function in Depressive Disorders: Insights, Challenges and Future Directions

**DOI:** 10.14336/AD.2024.0326

**Published:** 2024-03-26

**Authors:** Yuling Zhang, Chaozhi Tang

**Affiliations:** College of Life Sciences, Henan Normal University, Xinxiang, 453007, China.

**Keywords:** depression, microglia, neuroinflammation, heterogeneity, multi-omics, clinical translation

## Abstract

Microglia are crucial immune cells found in the central nervous system. Multiple investigations have substantiated the correlation between the development of depression and neuroinflammation resulting from impaired microglial activity. Through extensive research on the phenotype, function, imaging technology, multi-omics analysis, and in vitro culture of microglia in depressive disorder, the understanding of the relationship between microglia and depression has become more intricate. Various therapeutic approaches have been suggested, but a thorough analysis of the obstacles to clinical application has not been conducted. This paper explores the innovative advancement of microglia detection technology, recent research findings on microglia identification and epigenetic modification, the variability of microglia in different conditions, the relationship between microglia dysfunction and the onset of depression, the progress and challenges of microglia-targeted therapy for depression, and the current obstacles and future prospects in studying dysregulated microglial function in depressive disorders.

## 1. Introduction

Depression is the fourth largest contributor to global disease burden, with approximately 1/4 of women and 1/6 of men suffering from depression to varying degrees during their lifetime [1]. Depressed patients typically exhibit a diminished mood and a lack of interest. In more extreme instances, individuals may have symptoms of psychiatric conditions, including lack of focus, cognitive decline, heightened anxiety, intense dread, profound sadness, feelings of hopelessness, and inclinations towards self-harm [2]. The monoamine transmitter hypothesis is a well-established idea that posits the impaired activities of neurotransmitters such as 5-hydroxytryptamine (5-HT) and dopamine as the primary cause of depression. Antidepressants that target serotonin reuptake inhibitors, such as fluoxetine, paroxetine, sertraline, fluvoxamine, citalopram, and escitalopram, are the primary medications currently used for treating depression. However, only one-third of patients experience significant improvement in their depressive symptoms after taking these drugs, and there is a high risk of relapse after discontinuing the medication (approximately 62% for fluoxetine). Furthermore, prolonged usage of these medications will result in adverse effects such as nausea and diarrhea, sleeplessness, sexual dysfunction, and drug addiction [3, 4]. Studying novel pathophysiology and antidepressants is essential for effectively treating depression. Studies has discovered a correlation between depression and an elevation in the degree of inflammation in the neurological system [5-7]. The elevation of inflammation in the neurological system hinders the formation of new synaptic connections among neurons and the efficient transmission of neural signals, resulting in the impairment or dysfunction of neuronal activity [8]. Severe depression in patients is associated with the deterioration of brain networks involved in emotional regulation and the shrinking of important cognitive areas in the brain, such as the hippocampus [9-11]. Examining the correlation between inflammatory activity and depression has emerged as a recent area of interest.

Microglia are innate immune cells of the central nervous system, accounting for about 10% of the total number of brain cells [12]. Typically, microglia have the ability to efficiently eliminate dying cells and fragments by extending and retracting their processes and surveying the nearby molecular and cellular microenvironment [13, 14]. This process is crucial for maintaining the immune balance in the brain. The phagocytic activity of microglia is increased when activated by certain cytokines, lipopolysaccharide (LPS), toxic substances, chronic stress, or abnormal pressure. This activation leads to the production of inflammatory factors such as tumor necrosis factor α (TNF-α), interleukin-1 β (IL-1β), and IL-6. These factors can cause excessive synaptic pruning and potentially severe damage to neurons [4, 15-21].

Furthermore, when microglia are excessively activated, tryptophan, which is essential for the production of 5-HT, enters the kynurenine metabolic pathway. This results in the production of neurotoxic quinolinic acid, which in turn reduces the synthesis of 5-HT. Ultimately, this leads to the development of depression [22, 23].

Research has also discovered that some stimulating stimuli, such as alcohol, can induce the programmed cell death of microglial cells in the hippocampus. This can impair the ability of the hippocampal tissue to remove waste, result in malfunctioning of the hippocampal neurons, and give rise to behaviors depression-like [18]. These evidences indicate that malfunctioning microglia may play a crucial role in the onset of depression. However, the precise mechanisms by which microglial activation leads to depression are not yet completely comprehended.

This article explores the most recent advancements in microglia detection technology, the latest research findings on microglia identification and epigenetic modification, the variability of microglia in different conditions, the connection between microglia dysfunction and the onset of depression, the causal relationship, the progress in research, and the challenges in translating microglia-targeted therapy for depression into clinical practice. Proposed are challenges and future objectives for the study of the correlation between microglia and depression. An in-depth examination of these investigations will enhance our existing comprehension of dysregulated microglial activity in depressive disorders.

## 2. Two recent breakthroughs in the field of microglial research

### 2.1 Novel methodologies for investigating microglial function

#### 2.1.1 Single-cell transcriptomics and its examination of microglial heterogeneity

Microglia are dispersed heterogeneously throughout the central nervous system, with variations in their distribution across various brain areas. Furthermore, the morphology of microglia varies owing to variances in neuronal cell bodies, dendrites, axons, myelin sheaths, and the presence of blood vessels. Various physiological states or stimulation circumstances can elicit diverse reactions from distinct subsets of microglia. The phenotypic, cytokines, self-renewal, and transformation of microglia undergo dynamic changes in response to the body's state [24-26]. This phenomenon is referred to as microglia heterogeneity. Gaining insight into the diversity of microglia in the context of depression is crucial for comprehending microglia malfunction and devising precise therapeutic approaches.

Single-cell transcriptome analysis has been achieved by the advancement of flow cytometry, Qualcomm RNA-sequence detection, and microarray technology. This technology enables the separate analysis of the transcriptomes of different cells within the same tissue. It provides information about the transcription of various cell subsets in the tissue and their proportions. As a result, it can more accurately reveal the characteristic changes of small cell subsets during the development of a disease. Yaqubi et al. performed an extensive scRNA-seq investigation on the transcription patterns of human microglia at various ages. They discovered that microglia exhibited distinct transcriptional features to adapt to the dynamic brain microenvironment during the whole developmental process [27]. A separate analysis of gene expression in microglia from patients with major depressive disorder (MDD) revealed that 81 genes, such as CD163, MKI67, SPP1, CD14, FCGR1A/C, and C1QA/B/C, were found to be downregulated in the microglia of the occipital cortex. This down-regulation indicates a decrease in the immune function and phagocytic activity of microglia in individuals with MDD. CD47 and CD200 are involved in the transmission of signals from neurons to microglia, resulting in the inhibition of the microglial immune response and the prevention of synaptic pruning. The upregulation of CD200 and CD47 genes and proteins in the microglia of patients with MDD indicates that the immunosuppressive characteristics of microglia in MDD patients may be influenced by their own neuronal regulation [28]. A recent study analyzed snRNA-seq data from over 160,000 cells in the dorsolateral prefrontal cortex of 71 individuals, both male and female. The study revealed that in female patients with MDD, the majority of differentially expressed genes were found in microglia and parvalbumin interneurons (PV interneurons). Furthermore, the study observed abnormal expression of the SLIT-ROBO signal transduction pathway in female MDD patients, which plays a role in regulating the migration of microglia to specific neurons. The findings indicate that the development of female MDD is not just linked to the inflammatory reaction of microglia but also to the compromised interaction between microglia and PV interneurons [29]. This study addressed the lack of information on gender disparities in depression-related molecules by investigating gender-specific brain transcriptional groups associated with MDD in human patients.

The utilization of Sc/RNA-seq enables the identification of alterations in the transcriptome at the level of individual cells, therefore representing a significant advancement in our investigation of microglia heterogeneity. Nevertheless, the disintegration of tissues prior to sequencing and the subsequent absence of spatial data makes it impossible to comprehend the cellular interactions within the actual microenvironment. As a result, several scientists have suggested and developed spatial transcriptomics, a method that examines the transcriptome in space to discover the positional properties of the in-situ transcriptome. Wu et al. discovered a substantial correlation between microglia of the Mic03 subgroup and sadness by combining geographical and single-cell transcriptome data. This subgroup has elevated expressions of IQGAP2, FYN, PDE7A, ARHGEF3, and other genes that play a role in controlling microglia activation and neuroinflammation. Additionally, this subgroup demonstrates pronounced pro-inflammatory traits. The subgroup was designated as "pro-inflammatory microglia of depressive-like phenotypes" (PIMID). Upon conducting a more in-depth examination of the spatial transcriptome of the slice samples taken from the dorsal prefrontal cortex of the monkey brain, it was concluded that PIMID predominantly resides in the sixth layer of the monkey brain. This finding implies that targeted therapy aimed at these specific microglia could potentially serve as an effective approach to addressing depression [30].

#### 2.1.2 Utilizing sophisticated imaging methods to observe and track microglial activity in real-time

Obtaining detailed images of the rapid changes in microglia is essential for comprehending the functioning of the brain and its disorders. Using the CDr20 high-performance fluorescent chemical probe to identify microglia in vivo and in vitro is a straightforward process. Genomic CRISPR-Cas9 deletion screening revealed that UDP-glucuronosyl transferase Ugt1a7c acts as the binding molecule for CDr20. Furthermore, Ugt1a7c is particularly localized in microglia, allowing it to be anchored by fluorescence-labelled CDr20 [31]. Qin et al. recently published a study on a new in vivo adaptive optical three-photon microscopic imaging (AO-3PM) system. This system can accurately cause laser damage to the cerebral cortex of mice without damaging the skull. It can also clearly observe the entire process of microglial processes extending and wrapping around the laser damage points at a microscopic level [32]. This technique enables the actual monitoring of the dynamic changes of microglia in living organisms under specified situations and advances the progress of real-time monitoring of microglia's dynamic changes using high-resolution and large-field imaging technology.

The introduction of the MorphOMICs analysis technique has provided a fresh approach to describing the morphological alterations of microglia. MorphOMIC is a technique for topological data analysis that was created by Colombo et al. The method described in this study allows for the mapping of microglia morphology into multiple phenotypic maps that are related to clues. This method avoids the oversimplified categorization of microglia as either a multi-branched type in a surveillance state or an amoeba type in a phagocytic state. The result is a new method and data platform that can be used for more detailed studies on the heterogeneity of microglia [33].

#### 2.1.3 Development of microglia research models

The establishment of a research model for microglia is highly significant for comprehending the role of microglia in the central nervous system, the correlation between microglia and the onset of neurodegenerative disorders like depression, the identification and creation of specific drugs, and the assessment of the impact of environmental factors on the nervous system. Primary microglia are obtained directly from organisms without undergoing long-term passage or culture. These microglia are quite genuine and offer us the convenience to investigate their morphology, function, and mechanism. Inducible multifunctional stem cells, also known as induced pluripotent stem cells (iPSCs), address the ethical and source restrictions associated with primary microglia. Microglia may be readily generated and differentiated by manipulating the culture conditions and introducing certain stimuli. McQuade et al. effectively stimulated hematopoietic progenitor cells to differentiate into microglia by using cytokines such as M-CSF, IL-34, and TGF-β-1. This approach yielded a reliable cellular model for studying microglia in a laboratory setting [34].

Nevertheless, the study of individual microglia is now constrained by certain restrictions. However, the development of a range of three-dimensional brain-organ-like technologies offers a more authentic research model for mimicking the involvement of microglia in the development of intricate brain illnesses. Xu et al. generated neural progenitor cells (NPC) and primitive macrophage progenitors (PMP) from human pluripotent stem cells (hPSCs). They then co-cultured NPC and PMP to create brain organ-like structures that included microglia. A brain-like organ composed of human pluripotent stem cells (hPSCs) was created with the ability to adjust the ratio of microglia in order to mimic brain development and pathology [35]. The majority of brain organoids produced by neuroectodermal differentiation lack mesodermal cells that undergo development into microglia. Therefore, Professor Cakir's team at Yale University stimulated the production of functional microglia by activating the transcription factor PU.1 in human neuroectodermal organ-like organs (hCOs). These microglia are capable of safeguarding neurons from the harmful effects of Aβ deposition at a cellular and molecular level. This model provides a valuable opportunity to investigate the involvement of microglia in neurodevelopment and neurodegenerative disorders at the organ level in vitro [36]. A recent study published in Nature suggests that microglia may be generated by introducing primitive macrophages formed from human-induced pluripotent stem cells (iPSCs) into brain organoids obtained from the same origin. It has the identical PLIN2+ phenotype as embryonic microglia in mice and humans. This phenotype has the ability to restrict the proliferation of neural progenitor cells (NPCs), control the differentiation of NPCs, and facilitate the growth of axons [37].

Schafer et al. created an in vivo neuroimmune organoid model to more accurately replicate the life conditions of human microglia (hMGs) and assess the interaction and reaction between hMGs and the human brain environment. They utilized their newly discovered allogeneic transplantation technique to cultivate human Müller glia (hMGs) produced from human pluripotent stem cells (hPSCs) within vascularized human brain organoids in natural physiological settings in a living organism. Transcriptome sequencing revealed that hMGs possess transcriptome features that closely resemble those of microglia in the body. Using two-photon imaging technology, researchers discovered that hMGs play an active role in monitoring the brain environment that is similar to humans and may respond to harm caused by the environment [38, 39].

**Figure 1.**
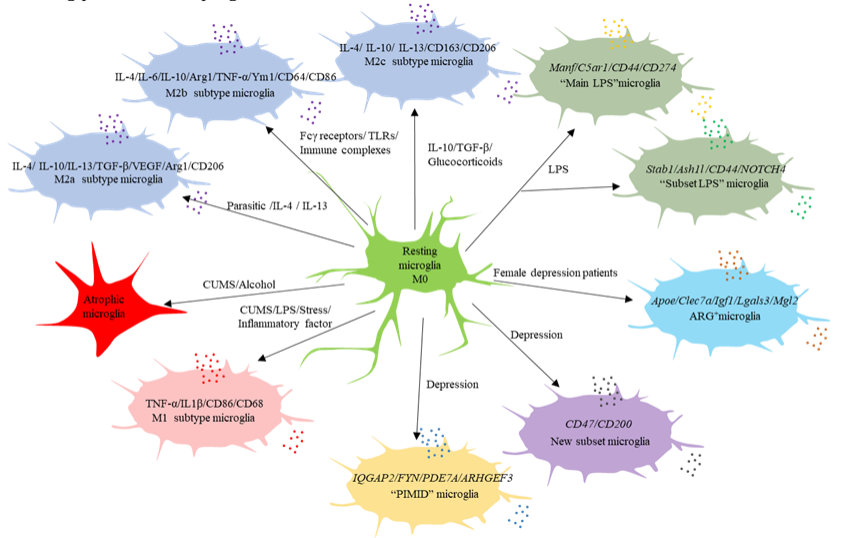
Identification of novel microglial activation states.

### 2.2 Significant discoveries from recent research

#### 2.2.1 Discovery of new microglial activation states

The activation of microglia is strongly linked to the disturbance of immunological balance in brain tissue. The detection of microglia activation has always been a central area of investigation in the field of neurodevelopment and brain disorders. In 1997, researchers initially detected "activated" microglia in vivo in regions of demyelination in two individuals diagnosed with multiple sclerosis [40]. To analyze the characteristics and activities of microglia in various settings, microglia in their normal state are referred to as the "resting state" or M0 phenotype. During pathological situations, microglia undergo a transition from a state of inactivity to a state of activation. They then polarize into either a typical pro-inflammatory state (M1 phenotype) or an alternate anti-inflammatory state (M2 phenotype) [41-43]. Among these, M1 microglia exhibited significant levels of expression for TNF-α, IL-1β, IFN-γ, and NO, as well as cell surface markers CD86 and CD68, among others. M2 microglia have significant levels of expression for IL-4, ARG1, Ym1, CD206, IL-1, and other related markers[42, 44]. M2 microglia may be classified into three distinct subtypes, namely M2a, M2b, and M2c, based on variations in gene expression profile and activation state (Fig. 1). M2a activation is triggered by parasites, IL-4, IL-13, and other signaling molecules and has a prolonged role in promoting resolution and repair. The M2b subtype can be generated by inducing polarization of the Fcγ receptor, TLR, and immune complex. M2c polarization is triggered by certain anti-inflammatory molecules, including IL-10, TGF-β, and glucocorticoid [45].

Recent investigations in single-cell transcriptomics have shown distinctive behavioural traits of microglia produced by inflammation. Sousa discovered that microglia activated by LPS exhibit distinct subsets. The "main LPS" microglia subset shows high expression of the Manf and C5ar1 genes, which are involved in promoting neuroprotection and tissue repair. On the other hand, the "subset LPS" microglia subset demonstrates high expression of the Stab1 and Ash1l genes, which suppress the production of pro-inflammatory mediators like IL-6 and TNF. Both microglia subsets express the CD44 membrane marker, while only the "main LPS" microglia subset expresses CD274, and the "subset LPS" microglia subset expresses NOTCH4 (See Fig. 1 for a visual representation). In comparison to the collection of microglia known as "main LPS," the genes associated with innate immunity and complement activation in the microglia subset known as "subset LPS" are downregulated and show less noticeable activity. These discoveries may offer fresh insights into the stimulation and recognition of microglia [46].

Furthermore, Stratoulias et al. discovered a microglia phenotype characterized by the presence of ARG1+ and high expression of Apoe, Clec7a, Igf1, Lgals3, and Mgl2 genes in the mouse brain (Fig. 1). This particular phenotype of microglia played a role in the development of the mouse brain and exhibited intriguing coincidences in both time and space with the cholinergic system. The absence of microglia in mice results in a decline in spatial memory and the connection between nerve cells that release acetylcholine. This work presents a promising new perspective for the advancement of microglial heterogeneity [47].

Recently found microglial subgroups may offer additional insights into identifying microglial activation. Scheepstra et al. discovered a new type of inhibitory microglia in the grey matter of the occipital cortex in female patients with major depressive disorder. This new phenotype was characterized by decreased expression of CD163, MKI67, SPP1, CD14, FCGR1A/C, and C1QA/B/C, and increased expression of CD47 and CD200 [28]. Wu and colleagues, using spatial transcriptome and single cell transcriptome data, found that a specific subset of microglia called Mic03 exhibited high expression of IQGAP2, FYN, PDE7A, and ARHGEF3 genes, which are involved in regulating microglial activation and neuroinflammation. These Mic03 microglia subsets were strongly associated with depression and showed significant pro-inflammatory properties [30], as depicted in Figure 1. The ongoing nature of these studies has significant significance in uncovering the intricacy of microglia activation and recognition.

#### 2.2.2 Epigenetic modifications in microglia and their relevance to depression

The investigation into the epigenetic alteration that controls the functioning of microglia is becoming more profound. In this section, we will provide a concise overview of significant discoveries pertaining to depression. Histone deacetylases (HDACs) are crucial regulators of epigenetic processes. HDAC11 is the sole member of the IV-type HDAC family and is predominantly found in the brain. Baek et al. discovered that the control of HDAC11 specifically triggers autophagy and maintains an active nitrogen balance in microglia. HDAC11 has the ability to alleviate depression by decreasing the activation of microglia [48]. Rodriguez-Zas et al. discovered that the signal route of histone acetylation in microglia was impaired in mice; exhibiting signs of depression-like behavior linked with inflammation [49]. EZH2, an enhancer of zeste homologue 2, is an enzyme that adds a methyl group to lysine 27 on histone H3. It has a role in controlling the activation of microglia, a kind of immune cell in the brain [50, 51]. Wang et al. discovered that chronic unpredictable mild stress (CUMS) leads to an upregulation of EZH2 expression in the prefrontal cortex and hippocampus of mice. This is followed by the activation of M1 microglia and an increase in the production of inflammatory markers. EZH2 inhibitors can alleviate cognitive impairment and depression-like behavior produced by CUMS by suppressing microglial activation and the production of proinflammatory cytokines [50]. Huang et al.'s study demonstrated that EZH2 had higher levels of expression in rats treated with CUMS and microglia activated by LPS. Following the suppression of EZH2, microglia underwent polarization towards the M2 phenotype, resulting in the reversal of depressive-like behaviour in the rat model. It was hypothesized that EZH2 hindered the production of miR-29b-3p by attaching to the miR-29b-3p promoter and enhancing histone H3-lysine 27-trimethylation. This, in turn, led to an increase in MMP2 transcription and the activation of M1-type polarization in microglia. The involvement of microRNA (miR)-146a in the regulation of innate immunity and inflammatory responses is well established; however, its contribution to the development of depression remains a subject of debate [51]. Liu et al. assert that upregulation of miR-146a can mitigate the depressive-like behavior induced by chronic unpredictable mild stress (CUMS) and lipopolysaccharide (LPS) injection by suppressing the activation of microglia [52]. Nevertheless, Fan et al. demonstrated that chronic unpredictable mild stress (CUMS) can upregulate the expression of miR-146a-5p in microglia. MiR-146a-5p inhibits neurogenesis and the spontaneous firing of excitatory neurons by targeting Krüppel-like factor 4 (KLF4) in the exocrine form, leading to depression-like behavior [53]. Circular RNA is intricately associated with the activation of microglia and the manifestation of depression. In their study, Yao et al. demonstrated that circular RNA DYM (circDYM) has the ability to control the activation of microglia by targeting miR-9 via HSP90 ubiquitin. Additionally, circDYM can also decrease microglial apoptosis by acting on the CEBPB/ZC3H4 axis. Furthermore, it was seen that circDYM may ameliorate depression-like behavior generated by LPS in mice [54, 55]. While there have been studies on the involvement of histone modification, microRNA, and circular RNA in controlling the activity of microglia in relation to depression, the depth of study on this topic is insufficient. Due to the presence of several epigenetic regulatory systems in the body, these systems may be interconnected to form a network that controls various physiological and pathological processes. Hence, it is important to thoroughly contemplate the impact of different epigenetic alterations on microglia and the development of depression.

## 3 Challenges in Microglial Research in the Context of Depression

### 3.1 Microglial response heterogeneity

Typically, microglia exhibit a balanced and adaptable state of stability and uniformity. However, in the presence of disease or abnormal conditions, this trait is readily disrupted, causing microglia that were previously in a state of rest to quickly transition into an activated state. Presently, the microglia at rest are referred to as M0 types and exhibit extensive branching. Conversely, activated microglia are primarily categorized into M1 and M2 types, characterized by larger cell size, a rounded cell body, and decreased branching. M1 microglia are a specific type of cell that primarily releases pro-inflammatory factors, which serve to promote inflammation. They stimulate the production of inflammatory mediators such as TNF-α, IL-1β, IL-6, glutamic acid, nitric oxide (NO), peroxides, and active free radicals. This leads to the initiation of an inflammatory response, resulting in apoptosis, secondary damage, and a significant neurotoxic effect. M2-type microglia can be categorized into three subtypes: M2a, M2b, and M2c. These subtypes secrete a significant amount of vascular endothelial growth factor (VEGF), transforming growth factor-β (TGF-β), IL-10, and other cytokines. These substances are crucial in promoting angiogenesis and the secretion of anti-inflammatory factors, which aid in the process of inflammatory repair [56, 57]. Neurodegenerative disorders, such as those characterized by aberrant gene expression, chronic unpredictable mild stress (CUMS), social failure, lipopolysaccharide (LPS) exposure, vaccination, brain damage, and Alzheimer's disease (AD), can induce depression-like behavior in animal models or people. This behavior is often associated with neuroinflammation, which is induced by the activation of microglia [19, 20, 58-62].

### 3.2 Causality and directionality

**Figure 2.**
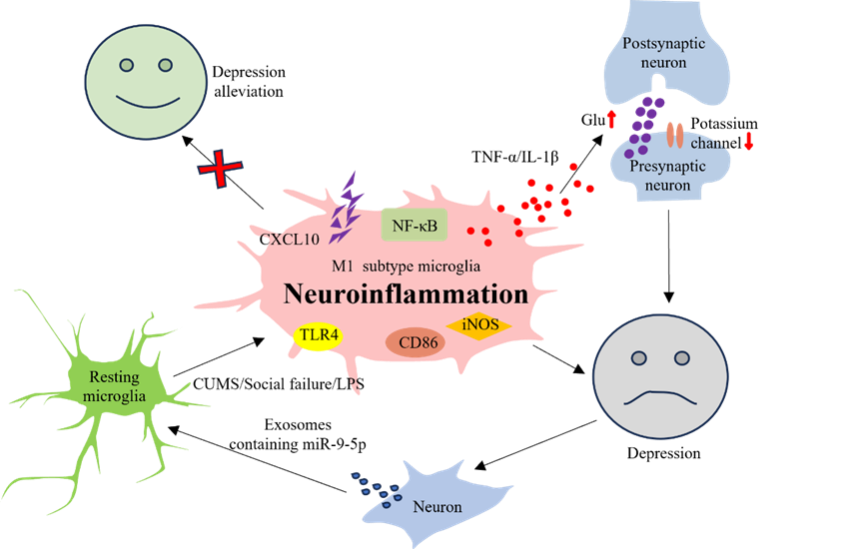
**The causal link between depression, microglial activation, and neuroinflammation**. Chronic unpredictable mild stress (CUMS), social isolation, and other stimulating events might induce the polarization of microglia towards the M1 phenotype, which is associated with the development of depression. Targeted elimination of microglia can reduce symptoms of depression. Furthermore, M1 phenotypic microglia can contribute to the development of depression by generating inflammatory substances like TNF-α and IL-1 β, which result in the atypical release of neurotransmitters like glutamate and the impairment of small-conductance calcium-activated potassium channel production and function. Neurons in individuals with depression can exacerbate depression by producing exocrine bodies that contain miR-9-5p, which promotes the polarization of microglia into the M1 phenotypic state.

The alteration of the microglia phenotype is intricately linked to the genetic variety of animal models or humans with depression and their surrounding environment. Yang et al. demonstrated that prolonged exposure to stress in female mice resulted in enduring depressive behavior. They also observed that microglia in the medial prefrontal cortex (mPFC) of mice were stimulated to become M1-type, and there was an elevation in the levels of NF-κB, IL-1β, and TLR4 expression. The removal of TLR4 gene expression in microglia led to a considerable reduction in the production of IL-1β [58]. Kokkosis et al.'s study showed that recurrent social failure results in the polarization of microglia into the M1 type, as well as elevated expression of CD86, iNOS, TNF-α, CXCL10, and IL-1β. The depression-like behavior was alleviated with the targeted removal of microglia [19]. Zheng et al. discovered that the activation of microglia in the basolateral amygdala of mice was triggered by LPS. Neuroinflammation causes an increase in the release of glutamate in the basolateral amygdala and a decrease in the expression and function of small-conductance. The calcium-activated potassium channel causes an overabundance of neuronal excitement, resulting in anxiety-depression-like behavior [20]. Consequently, several investigations posit that the atypical activation of microglia, the alteration of their condition, and the subsequent neuroinflammation are the underlying factors behind depression [8, 19-21, 58, 63-68]. Nevertheless, Xian et al.'s research demonstrated that the release of extracellular vesicles containing miR-9-5p from neurons of depressed individuals stimulated the activation of M1-type microglia. In depressed mice, the increased expression of miR-9-5p, facilitated by adeno-associated virus (AAV), led to the polarization of microglia into the M1 phenotype, thereby worsening depressive symptoms [69]. Thus, we posit that the atypical stimulation and alteration in the characteristics of microglia are both the underlying factor for depression and the consequence of post-depression induction. Microglia are activated abnormally due to external stimulus, pressure, and gene mutation, leading to a disruption in their activation and balance, which ultimately results in neuroinflammation. Abnormal neuroinflammation disrupts the microenvironment of neurons, leading to neuronal damage or death under stressful conditions. The alteration in neuronal state will unavoidably impact individual behavior and cognition, hence eliciting behaviors like depression. Depression leads to disruptions in many bodily processes, causing changes in the phenotype and condition of microglia. These changes are necessary to cope with and reverse any disturbances in the normal functioning of the central nervous system. The complete process can result in lasting and irreversible harm to microglia, neurons, synaptic connections, and transmission (Fig. 2). Hence, identifying the crucial factor that controls the microglia linked to neuroinflammation and depression could disrupt the harmful cycle of neuroinflammation and depression. This discovery offers potential solutions to the prevailing challenges of high prevalence, relapse, and drug resistance in treatment-resistant depression.

### 3.3 Translational hurdles

While there is evidence linking microglia to neuroinflammation and depression in organoids, rodents, and non-human primates, it is necessary to do additional research to see if these results are applicable to the development of depression in humans [70]. Researchers have developed drug delivery systems that specifically target microglia in the central nervous system or prevent their activation in animal models of depression [71, 72]. However, there are currently only a limited number of studies that have identified reliable biomarkers of microglial dysfunction in certain human patients with depression [28, 73, 74]. Furthermore, the ethical constraints of clinical research, patients' psychological receptiveness to research, and patients' adherence provide significant obstacles to the translation of research findings from preclinical models to clinical implementations.

## 4. Future Directions in Microglial-Depression Research

### 4.1 Targeted therapeutics

#### 4.1.1 Advancements in immunomodulatory treatments for depression

Increasingly, experts are focusing on the correlation between depression and the immune system. Researchers have extensively studied the immune mechanism of depression in recent years. This includes investigating the inflammatory response in both the central and peripheral systems, examining changes in cytokine levels, and exploring the connection between the immune system and the hypothalamic-pituitary adrenal axis (HPA). Additionally, researchers have looked into the interaction between the immune system and the brain stem, which is connected to the gut through the vagus nerve and blood vessels. The results presented here establish a theoretical foundation for using immune modulation as a therapy for depression [4, 6, 9, 11, 14, 17, 23, 39, 50, 60, 64, 66, 75-78].

#### 4.1.2 Personalized medicine approaches based on microglial profiles

The immunological mechanism of depression is intricate and differs significantly across individuals. However, the investigation of this mechanism appears to be strongly linked to changes in microglia phenotype, aberrant activation, and imbalance [4, 18, 19, 21, 28, 30, 52, 57, 59]. Hence, the advancement of individualized medicinal strategies grounded in microglia traits offers a fresh outlook for the identification and management of depression. Through the identification, analysis, monitoring, and prediction of the function and activity of microglia, we can enhance our understanding of the development of depression and provide patients with more efficient therapy alternatives. Advancements in technology and extensive study are anticipated to lead to significant advancements in the treatment of neurological disorders, such as depression, through tailored medicinal approaches that focus on microglia.

### 4.2 Multi-disciplinary collaborations

Depression is a multifaceted illness that can be caused by several factors, including alterations in the neurological and immunological systems, genetic abnormalities, and aberrant microglia, which can trigger depression [79-82]. Neuroscientists primarily investigate the functioning of the nervous system. One crucial field of research in neuroscience is the examination of how microglia, which act as protectors of the brain, impact neuroinflammation and depression [79]. The immune system and the neurological system are intricately interconnected and have a significant impact on the onset and progression of depression. Immunologists have concentrated their research on examining the immunological function of microglia and their reactions and alterations in depression in order to uncover the connection between the immune system and depression. [80, 81]. Geneticists research the impact of genes on microglia activity and the development of depression. By examining gene variations linked to microglia function and vulnerability to depression, they can gain valuable knowledge about the genetic mechanisms contributing to depression and identify prospective targets for therapy [82]. Hence, to gain a comprehensive comprehension of microglial function in depression, it is imperative for neuroscientists, immunologists, and geneticists to collaborate closely. This collaboration should involve integrating the most recent advancements in microglial-depression research from various disciplines and collectively unravelling the enigmas surrounding microglial function in depression through the sharing of data and resources. This multidisciplinary partnership aims to enhance progress in preventing and treating depression and lay the foundation for the creation of novel treatment approaches.

### 4.3 Longitudinal and multi-omics studies

Monitoring the alterations in microglia activity associated with depression is crucial for capturing the dynamic nature of microglia responses in various states and comprehending their dynamic characteristics [29, 59, 61, 83-87]. Wingo et al. discovered 19 genes that contribute to depression by analyzing the data from genome-wide association studies (GWAS) of 50,019 individuals with depression. They also examined the results of a whole proteome association study (PWAS) of the dorsal prefrontal cortex of 376 individuals with depression. Among these genes, LMBRD1 showed a significant enrichment in microglia. A meta-analysis of findings from proteome-wide association studies (PWAS) revealed that 25 brain proteins were strongly associated with depression. Specifically, ATG7 and LRP4 were found to be substantially expressed in microglia. Additionally, it was discovered that 20 genes corresponding to these 25 proteins were not identified in prior genome-wide association studies (GWAS) [88]. Furthermore, the researchers identified several genes associated with depression, such as Cntn1, TREM2, and P2X7, using SNP (single nucleotide polymorphism) and GWAS association analysis. Experimental studies in molecular biology have verified that these genes are strongly associated with the activation of microglia and neuroinflammation [61, 83, 84]. Research on microglia epigenomics has demonstrated that the methylation of microglia genes is influenced by factors such as age, brain location, mood disorders, and other psychiatric illnesses. Additionally, microglia exhibit distinct methylation patterns in various neuropsychiatric conditions [86]. The transcriptional group findings and phenotypes of microglia in depression animal models or people with varying stress situations and genders exhibit significant variations [29, 59]. Hence, the incorporation of multi-omics data is an essential approach to comprehensively comprehending the phenotype and function of microglia in depression. By combining these several types of biological data, we may obtain a more complete understanding of microglia in depression and gain insights into the molecular processes underlying the alterations in microglia associated with depression. This not only elucidates the underlying causes of depression but also offers a specific objective and foundation for the development of novel therapeutic approaches. Hence, conducting a comprehensive investigation into the alterations in microglia associated with depression and integrating data from many omics approaches is a crucial avenue for advancing research on depression.

## 5. Ethical Considerations and Future Challenges

Efforts to control the malfunctioning of microglia, which is associated with depression, have shown promising advancements in studies involving cells and animals. This offers a hopeful outlook for the potential therapeutic therapy of depression. As microglial-depression research progresses, an increasing number of researchers are investigating the connection between developing technologies, neuroregulatory treatment, and microglia and nerve inflammation. Emerging technologies encompass advanced imaging technology capable of high-resolution dynamic imaging, induced pluripotent stem cells (iPS), organoid culture, genomics, transcriptomics, proteomics, and other related fields. Neuromodulation treatments encompass several techniques such as transcranial electrical stimulation, electroshock, repeated transcranial magnetic stimulation, magnetic shock, and others [29, 31-34, 36, 37, 59, 61, 75, 83-87]. Depressed individuals frequently have a pessimistic attitude towards therapy and have an unpredictable emotional state. Recruiting depressed volunteers for large-scale clinical trials is a challenging task. Based on doctors' observations, the quality and capability of accompanying relatives of depressed patients significantly influences the clinical treatment outcome. However, ensuring the consistency and coordination of this aspect is challenging, which poses a difficulty. The development of clinical treatment standards should rely on comprehensive and in-depth comparative investigation over a longer duration. Furthermore, it is important to morally prioritize and address the recurrent fluctuations in mental state and diminished tolerance that commonly occur in individuals undergoing therapeutic therapy for depression. The uneven baseline condition of clinical treatment, variations in treatment cycles, fluctuations in the stability of outcomes, and even interruptions in clinical treatment have posed challenges to our efforts to construct precise depression therapy. These unique circumstances have necessitated the development of clinical treatment strategies and the need for more cautious evaluation of the effectiveness of treatments for depressed patients. Establishing a microglia-targeted therapy program that is rapid, accurate, efficient, and cost-effective will aid in mitigating or overcoming the impact of these factors. Creating a more advanced human organoid disease model to investigate the control of microglia activity is a novel approach to enhance the dependability and effectiveness of clinical trials.

Furthermore, there are upcoming obstacles that include the necessity to enhance our comprehension of the function of microglia in depression and how to utilize these discoveries to create or enhance more efficacious therapies. In order to guarantee the logical, secure, and fair utilization of these technologies, it is imperative to concentrate on deficiencies in ethical norms and the accessibility to state-of-the-art remedies for beneficiaries. This encompasses the task of ensuring that research adheres to ethical review prerequisites, guarantees the privacy and security of data, and safeguards the rights and interests of participants. Furthermore, it is crucial to thoroughly evaluate the possible hazards and advantages of novel technologies and therapies and guarantee that they undergo sufficient validation and scrutiny prior to implementation.

## 6. Conclusion

The involvement of microglia in depression is now a highly active subject of research in the fields of neuroscience and psychiatry. A comprehensive investigation and comprehension of the function of microglia in depression, followed by the formulation of novel therapy approaches and pharmacological targets, have the potential to significantly transform the management of treatment-resistant depression. Significant progress has been achieved in the fields of microglia omics, observation methods, heterogeneity analysis, and the integration of epigenetics. These advancements have helped to elucidate certain characteristics and patterns of microglia dysfunction during the course of depression. Furthermore, they have provided valuable insights for further comprehending the connection between microglia and depression. In light of this, future research will focus on investigating the activity of different microglia and finding ways to specifically regulate them, with the aim of advancing the treatment of depression. Considering the ethical implications, it is important to take into account the unique characteristics of individuals with depression and create treatment regimens that are more precise and effective in the practical implementation of microglia therapy.
